# Enhanced Electromagnetic Interference Shielding Properties of CNT/Carbon Composites by Designing a Hierarchical Porous Structure

**DOI:** 10.3390/nano14131099

**Published:** 2024-06-26

**Authors:** Yingying Yu, Yaxi Zhang, Yurong Zhou, Jiajia Xia, Minghui Chen, Huli Fu, Yufang Cao, Tao Wang, Cao Wu, Zhenmin Luo, Yongyi Zhang

**Affiliations:** 1 College of Safety Science and Engineering, Xi’an University of Science and Technology, Xi’an 710054, China; yyyu2022@xust.edu.cn (Y.Y.); komoriyui_1152@outlook.com (Y.Z.); christfer@xust.edu.cn (T.W.); 2 Key Laboratory of Multifunctional Nanomaterials and Smart Systems, Advanced Materials Division, Suzhou Institute of Nano-Tech and Nano-Bionics, Chinese Academy of Sciences, Suzhou 215123, China; yrzhou2017@sinano.ac.cn (Y.Z.); jjxia2021@sinano.ac.cn (J.X.); hlfu2017@sinano.ac.cn (H.F.); cwu2023@ahut.edu.cn (C.W.); yyzhang2011@sinano.ac.cn (Y.Z.); 3 International Iberian Nanotechnology Laboratory (INL), Avenida Mestre Jose Veiga, 4715-330 Braga, Portugal; 4 Shaanxi Yuanfeng Textile Technology Research Co., Ltd., Xi’an 710038, China; cmh930213@163.com; 5 School of Materials Science and Engineering, Anhui University of Technology, Ma’an Shan 243002, China

**Keywords:** electromagnetic interference shielding, carbon nanotube, amorphous carbon, hierarchical structure, pore engineering

## Abstract

With the widespread use of electronic devices, electromagnetic interference (EMI) has become an increasingly severe issue, adversely affecting device performance and human health. Carbon nanotubes (CNTs) are recognized for their electrical conductivity, flexibility, and stability, making them promising candidates for EMI shielding applications. This research developed hierarchical porous-structured CNT/carbon composites for enhancing electromagnetic interference (EMI) shielding properties. Featuring a CNT film with nano-scale pores and an amorphous carbon layer with micro-scale pores, the CNT/carbon composites are strategically arranged to promote the penetration of EM waves into the composite’s interior and facilitate multiple reflections, thereby improving the EMI shielding performance. An impressive EMI shielding effectiveness of 61.4 dB was achieved by the CNT/carbon composites, marking a significant improvement over the 36.5 dB measured for the pristine CNT film. Owing to the micro pores in the amorphous carbon layer, a notable reduction in the reflection shielding efficiency (SER) but, concurrently, a substantial increase in the absorption shielding efficiency (SEA) compared with the pristine CNT film was realized in the composites. This study successfully validated the effectiveness of the hierarchical porous structure in enhancing the EMI shielding performance, providing a promising new strategy for the development of lightweight, flexible, and efficient EMI shielding materials.

## 1. Introduction

With the fast development of electronic devices, the accompanied electromagnetic (EM) pollution poses a great threat to human health, and electromagnetic interference (EMI) has an adverse impact on the normal operation of electronic devices [1,2]. EM shielding materials, which can protect devices from EM waves or prevent EM wave leakage, are, therefore, in urgent demand [3,4]. Due to their outstanding electrical conductivity, excellent flexibility, and thermal/chemical stability, as well as their lightweight characteristic for high specific properties, carbon-based materials have attracted great attention and are considered new-generation EMI shielding materials with features against which traditional metal shielding materials cannot compare [5,6,7,8].

Carbon nanotubes (CNTs) are one kind of representative carbon-based EMI shielding material, usually utilized as fillers to improve the EMI shielding properties of polymer-based composites [9,10,11]. It was reported that to make polymers fulfill the fundamental requirements for commercial EMI materials, a concentration of 5 wt% multi-walled CNTs or 15 wt% single-walled CNTs within the polymer matrix is necessary, ensuring an EMI shielding effectiveness (SE) of greater than 20 dB [12,13]. Apart from polymer-based materials, Zhou et al. suggested an improvement in the EMI shielding performance of graphene film through the incorporation of CNTs [14]. An introduction of 20 wt% CNTs into the graphene film led to a 54% increase in electrical conductivity and a 20% enhancement in EMI shielding effectiveness (SE). Although further increasing the fraction of conductive CNT fillers may enhance the EMI shielding performance, it poses challenges due to the aggregation of CNTs, which subsequently have a negative impact on the EMI shielding performance as well as the mechanical properties of the polymer-based CNT composites [8].

Except for being utilized as fillers, self-assemblies of CNTs, e.g., compact films and porous foams, have exhibited unique advantages and great potential for EMI shielding applications [15,16,17,18]. The EMI shielding performance of CNT assemblies can be easily modulated by loading with conductive or magnetic particles, which act as auxiliary components for EMI shielding [19,20,21]. For example, Wang et al. synthesized a carbon-based composite with a CNT framework and temperature-sensitive microsphere loading, which enhanced the electrical conductivity after a post-thermal treatment and therefore led to an enhanced EMI SE in the composite [22,23]. Fu et al. loaded magnetic NiCo nanoparticles on a carbon-based foam composed of reduced graphene oxide (RGO) and single-walled carbon nanotubes (SWNTs) with oriented pores to improve the EMI shielding performance with the assistance of magnetic loss. The as-prepared composites exhibited an exceptional EMI SE of 105 dB within the frequency range of 8.2–12.4 GHz [24]. Furthermore, the porous structure enables multiple reflections of EM waves within the shielding materials due to their inherent substantial interfaces, which play an important role in reducing the transmitted EM wave and, therefore, enhance the overall EMI shielding performance [25,26,27,28,29].

As is widely known, the EMI shielding performance can be improved by either enhancing the absorption inside the shielding materials or increasing the reflection at the surface [30]. However, the ideal case for EMI shielding is that the incident EM wave is mostly absorbed by the shielding material with almost no reflection since the reflected EM wave can cause secondary pollution to the environment. A previous study demonstrated that CNT foams with a high porosity of > 90% led to a relatively large fraction of transmitted EM waves, which is not desirable for EMI shielding materials [25]. Meanwhile, due to the significant difference between the impedances of air and CNTs, a dense CNT assembly will give rise to a severe EM wave reflection at the surface of the shielding material, so that the EM wave can hardly enter the material, and the unique feature of porous CNT shielding materials, i.e., multiple reflections inside each individual pore, cannot contribute to the EMI shielding performance. Thus, a desired EMI shielding material should meet the requirement that EM waves can easily enter and deplete the material interior by EM wave multiple reflections [31,32].

In this work, a hierarchical porous-structured CNT/carbon composite was designed and synthesized. The amorphous carbon layer with micro-scale pores effectively reduced the EM waves’ reflection at the surface of the composite and enabled the penetration of the EM waves into the interior. A relatively dense CNT film with nano-scale pores facilitated the multiple reflections of EM waves, and the absence of large-scale pores prevented the direct penetration of EM waves through the CNT film. Enhanced EMI shielding efficiency and temperature stability were achieved in the hierarchical porous-structured CNT/carbon composite. This work provides a facile but effective strategy to improve the EMI shielding performance of porous materials, which can have a profound influence on the future development of lightweight, flexible, and effective EMI shielding materials.

## 2. Experimental Section

### 2.1. Preparation of the CNT/C Composite Film

**Figure 1:**
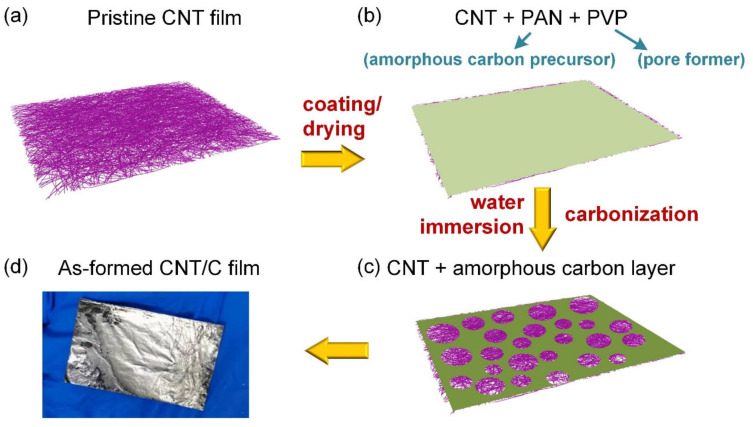
(**a**–**c**) Schematic of the preparation process of the hierarchical porous-structured CNT/C composites and (**d**) as-formed CNT/C film.

A schematic of the preparation of the hierarchical porous-structured CNT/carbon composites is depicted in Figure 1. The pristine carbon nanotube (CNT) films utilized in this study were supplied by JERNANO Co., Ltd., which were fabricated via a floating catalytic chemical vapor deposition (FC-CVD) process, with ethanol as the carbon source, ferrocene as the catalyst, and thiophene as the promoter. The reactant mixture was injected into a tubular furnace, which was maintained at a temperature of 1150 °C. The deposition occurred within a carrier gas environment consisting of a mixture of argon and hydrogen. This enabled the formation of a continuous carbon nanotube network structure. Subsequently, the CNT film was continuously collected at the furnace outlet, facilitating the production of a roll of CNT film [33]. As illustrated in Figure 1a, the pristine CNT film was constructed by entangled CNTs with a clear interspace between the adjacent CNTs, which could not effectively hinder the EM wave. The SEM image of the pristine CNT film is shown in Figure S1 of the Supplementary Materials, verifying an average interspace of a hundred nanometers between the CNTs.

To realize the hierarchical porous structure, an N,N-dimethylformamide (DMF) solution of 5 wt% polyacrylonitrile (PAN, the precursor of amorphous carbon) and polyvinyl pyrrolidone (PVP, the pore former) was prepared with different PVP concentrations (1.5 wt%, 2.5 wt%, 5 wt%, and 10 wt%, and the corresponding samples were denoted by CNT/C-1.5, CNT/C-2.5, CNT/C-5, and CNT/C-10, respectively). The as-prepared solution was applied to the surfaces of the CNT films, followed by a drying process in a vacuum oven to remove the DMF solvent. Then, the samples were immersed in water to separate the PVP and PAN phases, and micro-scale pores were formed with a homogeneous distribution after the dissolution of the PVP. The CNT/PAN composites were then treated with a pre-oxidation process at 220 °C, 250 °C, and 280 °C for 30 min, 30 min, and 60 min, respectively, and were finally carbonized at 900 °C for 60 min with a protective atmosphere of argon (Ar) to form the hierarchical porous-structured CNT/C composites. Moreover, the surfaces of the CNT/C composites presented a metallic luster and mirror-like surface quality, as depicted in Figure 1d. This indicated that the applied amorphous carbon layer was of low roughness.

### 2.2. Characterization of the CNT/C Composite Films

The microstructural analysis of the CNT/C composite films was conducted using a scanning electron microscope (SEM, model Hitachi S-4800) operated at an acceleration voltage of 10 kV. The contact angle measurements were performed with an optical contact angle goniometer (OCA15EC, dataphysic, Germany). The electromagnetic interference (EMI) shielding effectiveness (SE) was evaluated using a vector network analyzer (N5227A, Agilent Technologies) within the frequency range of 8.2–12.4 GHz, employing the waveguide method. To determine the electrical conductivity, the specimens were cut into 1 mm × 20 mm strips and coated with silver electrodes. The electrical conductivities were then measured using a two-probe technique with a Keithley multimeter system (DAQ6510). Raman spectroscopy was carried out using a LabRAM ARAMIS Raman confocal microscope (HORIBA JobinYvon) with an excitation wavelength of 532 nm, and the spectra were captured with a 10-s integration time. Thermal stability was assessed by thermogravimetric analysis (TGA) using a NETZSCH TG 209 F1 Libra instrument, with a temperature range from 30 °C to 900 °C and a heating rate of 10 °C·min^−1^. The elemental composition of the films was determined by X-ray photoelectron spectroscopy (XPS, ESCALAB 250XI, Thermo Fisher Scientific).

## 3. Results and Discussion

**Figure 2:**
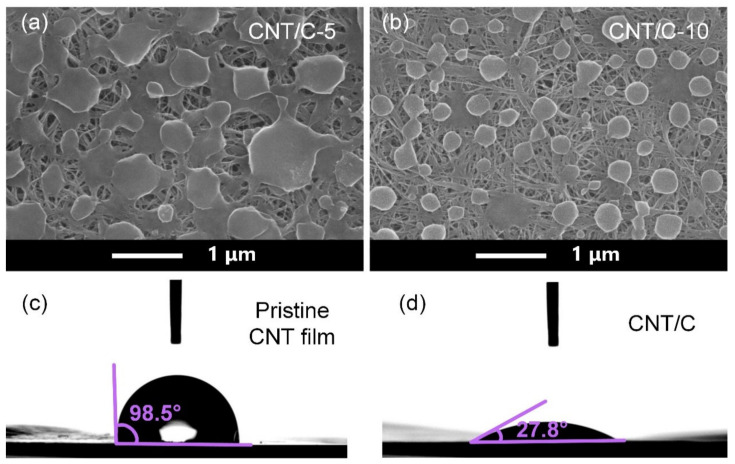
(**a**,**b**) SEM images of the CNT/C-5 and CNT/C-10 samples; (**c**,**d**) surface wettability of pristine CNT film and as-formed CNT/C film.

The morphology of the CNT/C composites was characterized by SEM, as depicted in Figure 2a,b. The hierarchical porous structure can be clearly observed in the SEM images, which consisted of nano-scale pores in the CNT film and micro-scale pores in the amorphous carbon layer. The size of the micro-scale pores could be well tuned by adjusting the PVP concentration in the coating concentration, for example, the CNT/C-5 sample featured micro-scale pores, while the CNT/C-10 sample had much larger pores, so the amorphous carbon phase was isolated. The dimensions of the as-formed “amorphous carbon island (ACI)” were statistically studied, and the average dimension of the ACI in CNT/C-5 was around 542 nm, while that in CNT/C-10 was around 311 nm. The hydrophilicity of the carbon films was tested by measuring the contact angle of water. Figure 2c,d show photographs of a water droplet standing on the surfaces of the pristine CNT film and the CNT/C film. A significant decrease in the water contact angle from 98.5° to 27.8° indicated a hydrophobic-to-hydrophilic transition on the sample surface by coating the porous carbon layer. This transition may limit the use of the CNT/C film for EMI shielding in humid environments.

**Figure 3:**
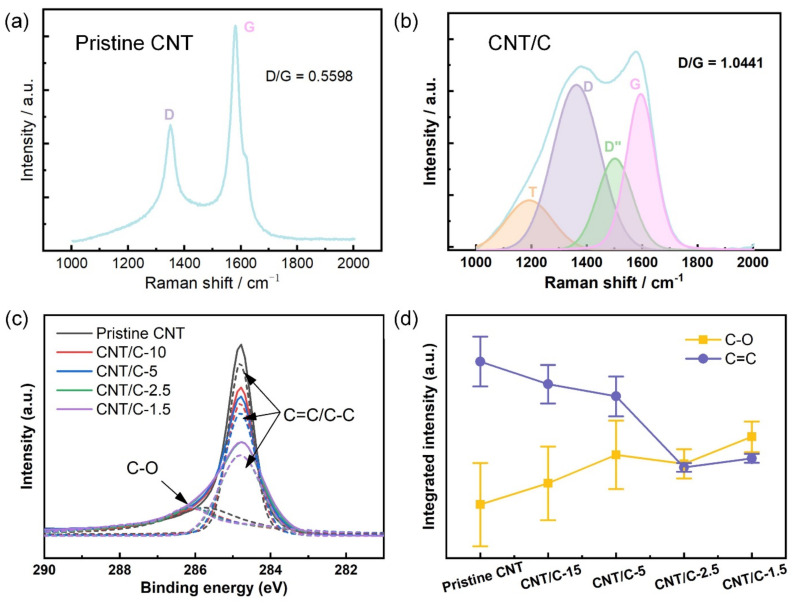
Raman spectra of (**a**) pristine CNT film and (**b**) CNT/C-x sample; (**c**) XPS spectra of pristine CNT film and CNT/C composites prepared with different PVP concentrations; (**d**) integrated intensity of the C-O and C=C/C-C peaks in the XPS spectra as a function of PVP concentration.

The chemical environment of the pristine CNT film and CNT/C composites was characterized by Raman spectroscopy and the XPS technique, as shown in Figure 3. In Raman spectra, the representative peaks T (~1230 cm^−1^), D (~1360 cm^−1^), D″ (~1470 cm^−1^), and G (~1570 cm^−1^) are typically observed for carbon materials, which correspond to the disordered graphitic carbon lattice, lattice defects of the carbon atom, amorphous carbon structure, and in-plane vibration of the carbon atoms, respectively [34]. As depicted in Figure 3a, only D and G peaks appear in the Raman spectrum of the pristine CNT film, indicating the absence of a disordered graphitic carbon lattice and amorphous carbon. Meanwhile, peaks T and D″ are present in the spectrum of the CNT/C sample, indicating the coated carbon layer is in amorphous form. The appearance of the disordered graphitic carbon lattice in the CNT/C sample may be attributed to the interface between the CNT film and carbon layer, where carbon atoms in the two layers are bonded by van der Waal’s forces, leading to a distortion of the graphitic lattice in the CNT. The intensity ratio between D and G is usually used as an indicator to quantify graphitization: the smaller the D/G, the higher the graphitization degree of the sample [35]. It can be noted that the D/G ratio increased in the CNT/C sample compared with the pristine CNT, indicating the decrease in the graphitization degree, which is because the introduction of amorphous carbon averages out the fraction of graphitized carbon atoms.

The XPS spectra in the energy range of 281 eV to 290 eV are depicted in Figure 3c. An asymmetric peak consisting of individual peaks at 284.8 eV and 285.6 eV, which can be ascribed to C=C/C-C and C-O, respectively [34], was observed in all the samples. Gaussian peak fitting was performed to investigate the intensity of the two individual peaks in the pristine CNT and CNT/C samples processed with different PVP concentrations. The integrated intensity of the C=C/C-C peak and C-O peak is shown in Figure 3d. It can be noted that the intensity of the C=C/C-C peak was highest in the pristine CNT sample, and it decreased in the CNT/C samples with a decreasing PVP concentration. The integrated intensity of the C-O peak exhibits an opposite trend to that of the C=C/C-C peak. In the pristine CNT, most of the carbon atoms were graphitic carbon atoms, giving rise to a strong C=C/C-C peak. The small and diffuse C-O peak may have arisen from a small fraction of oxidized carbon atoms. For the CNT/C composites, the samples processed with lower PVP concentrations had a smaller pore fraction, and their surfaces were more largely covered with amorphous carbon. Since the XPS result was more representative in characterizing the chemical bond on the sample surface, the samples covered with more amorphous carbon exposed less graphitic carbon atoms, leading to a weaker C=C/C-C. peak.

**Figure 4:**
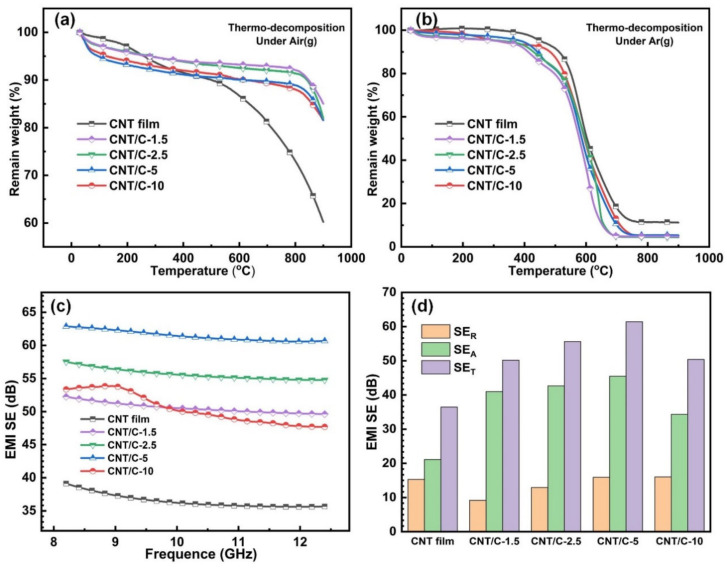
(**a**,**b**) TG results of pristine CNT film and CNT/C composites prepared with different PVP concentrations in atmospheres of the air and argon; (**c**,**d**) the electromagnetic interference shielding properties of pristine CNT film and CNT/C composites prepared with different PVP concentrations.

The thermal stability of the pristine CNT and CNT/C composites was examined by TG analysis. The weight percentage curves with increasing temperature in an argon and air atmosphere are depicted in Figure 4a,b. For the air atmosphere, all the samples were resistless to high temperatures, experiencing a dramatic weight percentage drop after ~550 °C, which should be mainly due to the oxidation of carbon. Note that in the early stage of heating (<400 °C), the CNT/C composites had larger weight loss than the pristine CNT for both the argon and air atmosphere, and this weight percentage difference was maintained after 800 °C in the air. This could be attributed to a decomposition of the unstable impurities in the amorphous carbon layer. In an argon atmosphere, the thermal stability of CNT/C composites was significantly enhanced compared with the pristine CNT, as indicated by the result that the weight percentage of the CNT/C composites remained at 85–95% up to 800 °C, whereas the weight percentage of the pristine CNT dropped to ~70%. The composite sample processed with a lower PVP concentration even had better thermal stability, which may be related to the larger covering area of the amorphous carbon. The EMI SE of the pristine CNT and CNT/C composites in the frequency range of 8.2–12.4 GHz are depicted in Figure 4c.

All the CNT/C composites exhibited an enhancement of EMI SE in the measuring frequency range, indicating the effectiveness of constructing the hierarchical porous structure for improving the EMI shielding performance. The pore size and the covering area of the amorphous carbon layer have an important influence on the EMI shielding performance. If the pore size is too small or the covering area is too large, the EM wave is largely blocked by the amorphous carbon layer and can hardly enter the CNT film. Meanwhile, an insufficient covering area of the amorphous carbon layer cannot effectively reduce the impedance and leads to an inferior impedance matching. It can be seen in Figure 4c that the CNT/C-5 sample had the highest EMI SE within the measured frequency range, which had the optimal balance between letting the EM wave enter the composite and impedance matching at the surface. To gain deeper insight into the EMI shielding mechanism of the CNT/C composite films, the total EMI shielding efficiency (*SE_T_*), the reflection shielding efficiency (*SE_R_*), and the absorption shielding efficiency (*SE_A_*) of the samples were calculated based on the waveguide method, where the *S*-parameters (*S*_11_, *S*_12_, *S*_21_, and *S*_22_) were obtained via a four-port vector network analyzer, as described in the Supplementary Information. First, the coefficients of reflectivity, transmissivity, and absorptivity, denoted by *R*, *T*, and *A*, respectively, can be calculated from *S*_11_ and *S*_21_ according to the following equations [8]:

*R* = |*S*_11_|^2^ (1)

*T* = |*S*_21_|^2^ (2)

*A* = 1 − *R* − *T* (3)

The effective absorptivity (A_eff_) can be described as:

*A*_eff_ = (1 − *R* − *T*)/(1 − *R*) (4)

Then, the *SE_T_* can be determined by the sum of the *SE_R_*, the *SE_A_*, and the multiple internal reflections (*SE_M_*) of electromagnetic radiation.

SE_T_ = SE_R_ + SE_A_ + SE_M_ (5)

When *SE_T_* > 15 dB, the SEM can be ignored, and the SE_T_ is usually defined as follows:

SE_T_ ≈ SE_R_ + SE_A_ (6)

where the *SE_R_* and *SE_A_* are calculated according to the following equations:

*SE_R_* = −log_10_(1 − *R*) (7)

*SE_A_* = −log_10_(1 − *A_eff_*) = −log_10_[*T*/(1 − *R*)] (8)

The as-calculated *SE_A_*, *SE_R_*, and *SE_T_* are summarized in Figure 4d. The *SE_R_* of the composite films was greatly decreased with the introduction of the less conductive amorphous carbon. Then, the *SE_R_* was slightly increased with an enhanced PVP concentration, owing to the larger pores that appeared on the surfaces of the composite films. The *SE_A_* presented an opposite tendency of the SET, which has a strong relationship with the number of inner heterogeneous interfaces between the conductive CNT and less conductive amorphous carbon. As a result, the CNT/C-5 possessed the highest EMI SE of 61.4 dB, which was much higher than that of the pristine CNT films of 36.5 dB. Overall, by designing a hierarchical pore structure, the EMI SE performance could be greatly increased, mainly because of the absorption of the EM waves, which is desirable for both EM wave blocking and environmental health.

## 4. Conclusions

In summary, this research successfully designed and synthesized a novel hierarchical porous-structured CNT/carbon composite that demonstrates remarkable enhancements in EMI shielding performance. The introduction of an amorphous carbon layer with micro-scale pores effectively reduced the surface reflection and allowed EM waves to penetrate into the interior of the relatively dense CNTs with nano-scale pores, where multiple reflections were facilitated. By tuning the structural features of the hierarchical pores in the CNTs and amorphous carbon, a balance between EM wave penetration and impedance matching was optimized. As a result, the composite achieved an impressive EMI shielding effectiveness of 61.4 dB, far surpassing the 36.5 dB measured for the pristine CNT film. This work not only provides a promising strategy for improving EMI shielding performance but also offers a new perspective for the development of advanced lightweight and flexible EMI shielding materials.

## Data Availability

The original contributions presented in the study are included in the article/Supplementary Materials.

## Supplementary Materials

The following supporting information can be downloaded at: https://www.mdpi.com/article/10.3390/nano14131099/s1, Figure S1: SEM image of the pristine CNT film; Figure S2: Schematic of the EMI SE characterization using waveguide method.

## References

[1] Grellier J., Ravazzani P., Cardis E. (2014). Potential health impacts of residential exposures to extremely low frequency magnetic fields in Europe. Environ. Int..

[2] Schüz J., Ahlbom A. (2008). Exposure to electromagnetic fields and the risk of childhood leukaemia: A review. Radiat. Prot. Dosim..

[3] Cheng J.Y., Li C.B., Xiong Y.F., Zhang H.B., Raza H., Ullah S., Wu J.Y., Zheng G.P., Cao Q., Zhang D.Q. (2022). Recent advances in design strategies and multifunctionality of flexible electromagnetic interference shielding materials. Nano-Micro Lett..

[4] Jia X.C., Li Y., Shen B., Zheng W.G. (2022). Evaluation, fabrication and dynamic performance regulation of green EMI-shielding materials with low reflectivity: A review. Compos. Part B: Eng..

[5] Wu G., Chen Y., Zhan H., Chen H.T., Lin J.H., Wang J.N., Wan L.Q., Huang F.R. (2020). Ultrathin and flexible carbon nanotube/polymer composite films with excellent mechanical strength and electromagnetic interference shielding. Carbon.

[6] Ameli A., Arjmand M., Pötschke P., Krause B., Sundararaj U. (2016). Effects of synthesis catalyst and temperature on broadband dielectric properties of nitrogen-doped carbon nanotube/polyvinylidene fluoride nanocomposites. Carbon.

[7] Yu Y.Y., Zhang L.W., Yildiz O., Deng H.T., Zhao C.H., Bradford P.D., Li J.Y., Zhu Y.T. (2017). Investigation of microcombing parameters in enhancing the properties of carbon nanotube yarns. Mater. Des..

[8] Gupta S., Tai N. (2019). Carbon materials and their composites for electromagnetic interference shielding effectiveness in X-band. Carbon.

[9] Wang Y.Y., Zhou Z.H., Zhou C.G., Sun W.J., Gao J.F., Dai K., Yan J.X., Li Z.M. (2020). Lightweight and robust carbon nanotube/polyimide foam for efficient and heat-resistant electromagnetic interference shielding and microwave absorption. ACS Appl. Mater. Interfaces.

[10] Song Q., Ye F., Yin X.W., Li W., Li H.J., Liu Y.S., Li K.Z., Xie K.Y., Li X.H., Fu Q.G. (2017). Carbon nanotube-multilayered graphene edge plane core-shell hybrid foams for ultrahigh-performance electromagnetic-interference shielding. Adv. Mater..

[11] Chen Y., Zhang H.B., Yang Y.B., Wang M., Cao A.Y., Yu Z.Z. (2016). High-performance epoxy nanocomposites reinforced with three-dimensional carbon nanotube sponge for electromagnetic interference shielding. Adv. Funct. Mater..

[12] Li N., Huang Y., Du F., He X.B., Lin X., Gao H.J., Ma Y.F., Li F.F., Chen Y.S., Eklund P.C. (2006). Electromagnetic interference (EMI) shielding of single-walled carbon nanotube epoxy composites. Nano Lett..

[13] Arjmand M., Apperley T., Okoniewski M., Sundararaj U. (2012). Comparative study of electromagnetic interference shielding properties of injection molded versus compression molded multi-walled carbon nanotube/polystyrene composites. Carbon.

[14] Zhou E., Xi J.B., Guo Y., Liu Y.J., Xu Z., Peng L., Gao W.W., Ying J., Chen Z.C., Gao C. (2018). Synergistic effect of graphene and carbon nanotube for high-performance electromagnetic interference shielding films. Carbon.

[15] Li M.Z., Jia L.C., Zhang X.P., Yan D.X., Zhang Q.C., Li Z.M. (2018). Robust carbon nanotube foam for efficient electromagnetic interference shielding and microwave absorption. J. Colloid Interface Sci..

[16] Han L.Y., Song Q., Li K.Z., Yin X.M., Sun J.J., Li H.J., Zhang F.P., Ren X.R., Wang X. (2021). Hierarchical, seamless, edge-rich nanocarbon hybrid foams for highly efficient electromagnetic-interference shielding. J. Mater. Sci. Technol..

[17] Lan C.T., Guo M., Li C.L., Qiu Y.P., Ma Y., Sun J.Q. (2020). Axial alignment of carbon nanotubes on fibers to enable highly conductive fabrics for electromagnetic interference shielding. ACS Appl. Mater. Interfaces.

[18] Wang T.X., Xiong C.Y., Zhang Y.K., Wang B., Xiong Q., Zhao M.J., Ni Y.H. (2024). Multi-layer hierarchical cellulose nanofibers/carbon nanotubes/vinasse activated carbon composite materials for supercapacitors and electromagnetic interference shielding. Nano Res..

[19] Zhao H.H., Ji K.J., Liu T.T., Xu Y.S., Dai Z.D. (2014). Electrophoretic deposition of foam Ni/CNT composites and their electromagnetic interference shielding performance. Appl. Mech. Mater..

[20] Çakmakçı N., Kim G., Song H., Shin M., Jung Y., Jeong Y. (2023). Ferrite-decorated ultrathin and lightweight carbon nanotube film for electromagnetic interference shielding. ACS Appl. Nano Mater..

[21] Chao Z., Yu Y.Y., Lei F., Hu D.M. (2021). A lightweight and flexible CNT/Fe3O4 composite with high electromagnetic interference shielding performance. Carbon Lett..

[22] Cai J.H., Li J., Chen X.D., Wang M. (2020). Multifunctional polydimethylsiloxane foam with multi-walled carbon nanotube and thermo-expandable microsphere for temperature sensing, microwave shielding and piezoresistive sensor. Chem. Eng. J..

[23] Cai J.H., Tang X.H., Chen X.D., Wang M. (2021). Temperature and strain-induced tunable electromagnetic interference shielding in polydimethylsiloxane/multi-walled carbon nanotube composites with temperature-sensitive microspheres. Composites Part A.

[24] Fu H.L., Chen L., Liu D.P., Zhang Y.Y., Cao Y.F., Wu C., Yong Z.Z., Yu Y.Y., Li Q.W. (2023). Multifunctional NiCo@RGO/SWNTs foam with oriented pore structure for excellent electromagnetic interference shielding. Chem. Eng. J..

[25] Yu Y.Y., Chao Z., Gong Q., Li C.W., Fu H.L., Lei F., Hu D.M., Zheng L.X. (2021). Tailoring hierarchical carbon nanotube cellular structure for electromagnetic interference shielding in extreme conditions. Mater. Des..

[26] Xue T.T., Yang Y., Yu D.Y., Wali Q., Wang Z.Y., Cao X.S., Fan W., Liu T.X. (2023). 3D printed integrated gradient-conductive MXene/CNT/polyimide aerogel frames for electromagnetic interference shielding with ultra-low reflection. Nano-Micro Lett..

[27] Zhan Y.H., Oliviero M., Wang J., Sorrentino A., Buonocore G.G., Sorrentino L., Lavorgna M., Xia H.S., Iannace S. (2019). Enhancing the EMI shielding of natural rubber-based supercritical CO2 foams by exploiting their porous morphology and CNT segregated networks. Nanoscale.

[28] Shen B., Li Y., Zhai W.T., Zheng W.G. (2016). Compressible Graphene-Coated Polymer Foams with Ultralow Density for Adjustable Electromagnetic Interference (EMI) Shielding. ACS Appl. Mater. Interfaces.

[29] Zeng Z.H., Jin H., Chen M.J., Li W.W., Zhou L.C., Zhang Z. (2016). Lightweight and Anisotropic Porous MWCNT/WPU Composites for Ultrahigh Performance Electromagnetic Interference Shielding. Adv. Funct. Mater..

[30] Wang H., Li S.N., Liu M.Y., Li J.H., Zhou X. (2021). Review on shielding mechanism and structural design of electromagnetic interference shielding composites. Macromol. Mater. Eng..

[31] Xu L., Jia L.C., Yan D.X., Ren P.G., Xu J.Z., Li Z.M. (2018). Efficient electromagnetic interference shielding of lightweight carbon nanotube/polyethylene composites via compression molding plus salt-leaching. RSC Adv..

[32] Zhang L.Q., Yang S.G., Li L., Yang B., Huang H.D., Yan D.X., Zhong G.J., Xu L., Li Z.M. (2018). Ultralight cellulose porous composites with manipulated porous structure and carbon nanotube distribution for promising electromagnetic interference shielding. ACS Appl. Mater. Interfaces.

[33] Xu H., Tong X., Zhang Y.Y., Li Q.W., Lu W.B. (2016). Mechanical and electrical properties of laminated composites containing continuous carbon nanotube film interleaves. Compos. Sci. Technol..

[34] Yang L., Chen G.L., Zhang N., Xu Y.X., Xu X.F. (2019). Sustainable biochar-based solar absorbers for high-performance solar-driven steam generation and water purification. ACS Sustain. Chem. Eng..

[35] Cançado L.G., Takai K., Enoki T., Endo M., Kim Y.A., Mizusaki H., Jorio A., Coelho L.N., Magalhães-Paniago R., Pimenta M.A. (2006). General equation for the determination of the crystallite size La of nanographite by Raman spectroscopy. Appl. Phys. Lett..

