# Supplementary material for: Enhanced Electromagnetic Interference Shielding Properties of CNT/Carbon Composites by Designing a Hierarchical Porous Structure
Source: Nanomaterials (Basel). 2024 Jun 26;14(13):1099. doi: 10.3390/nano14131099 (PMC11243308; doi:10.3390/nano14131099)
Supplement: Supplementary file 1 [file nanomaterials-14-01099-s001.zip › nanomaterials-3053137-supplementary.pdf]

Supplementary Material

Enhanced Electromagnetic Interference Shielding Properties of CNT/Carbon Composites by Designing a Hierarchical Porous Structure

Yingying Yu[1,2], Yaxi Zhang[1], Yurong Zhou[2,3], Jiajia Xia[2], Minghui Chen[4], Lin Liu[4], Huli Fu[2], Yufang Cao[2,*], Tao Wang[1], Cao Wu[2,5], Zhenmin Luo[1,*], Yongyi Zhang[2]

[1] College of Safety Science and Engineering, Xi'an University of Science and Technology, Xi'an 710054, Shaanxi, China;

[2] Key Laboratory of Multifunctional Nanomaterials and Smart Systems, Advanced Materials Division, Suzhou Institute of Nano-Tech and Nano-Bionics, Chinese Academy of Sciences, Suzhou 215123, Jiangsu, China;

[3] International Iberian Nanotechnology Laboratory (INL), Avenida Mestre Jose Veiga, Braga 4715-330, Portugal;

[4] School of Materials Science and Engineering, Anhui University of Technology, Ma'an Shan 243002, Anhui, China;

[5] Shaanxi Yuanfeng Textile Technology Research Co., Ltd, Xi'an 710038, Shaanxi, China;

* Corresponding authors: zmluo@xust.edu.cn (Z. Luo); yfcao2019@sinano.ac.cn (Y. Cao)

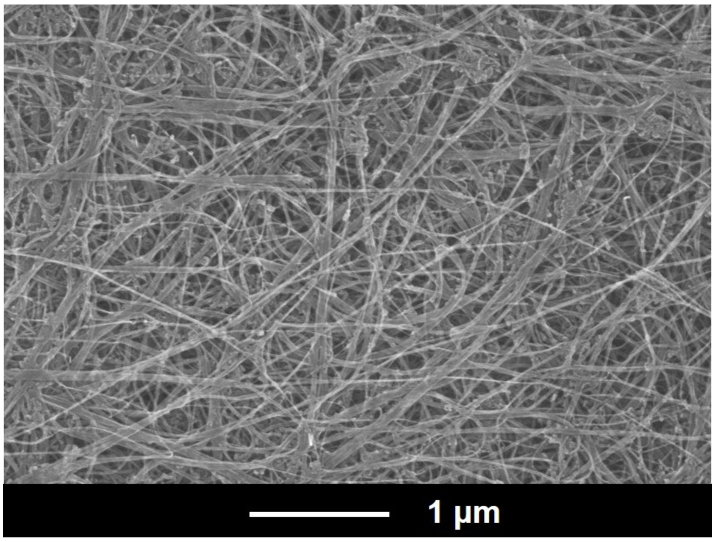

Figure S1 SEM image of the pristine CNT film

Characterization of electromagnetic interference (EMI) shielding effectiveness (SE) using waveguide method

The schematic of characterizing the EMI SE using waveguide method is depicted below.

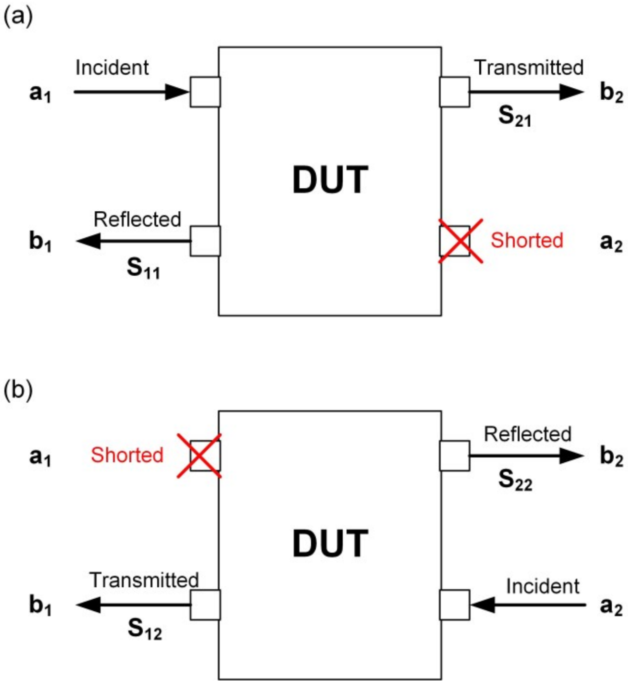

Figure S2. Schematic of the EMI SE characterization using waveguide method

The measurement was carried out with a four-port vector network analyzer. The measured sample (DUT) was inserted between the four ports, with two ports on each side. The voltages of the four ports are denoted by a_1, b_1, b_2 and a_2. The measurement was conducted with two configurations. First, voltage was inputted from the a_1 port and a_2 port was shorted so that a_2=0. The reflection and transmission coefficient S_{11} and S_{21} was calculated with the equations below:

$$S_{11} = \frac{b_1}{a_1}\Big|_{a_2=0} \tag{1}$$

$$S_{21} = \frac{b_2}{a_1}\Big|_{a_2=0} \tag{2}$$

Then, voltage was inputted from the port a_2 and a_1 was shorted. The reflection and transmission coefficient S_{22} and S_{12} was calculated with the equations below:

$$S_{22} = \frac{b_2}{a_2}\Big|_{a_1=0} \tag{3}$$

$$S_{12} = \frac{b_1}{a_2}\Big|_{a_1=0} \tag{4}$$

With the calculated S_{11}, S_{21}, S_{22} and S_{12} coefficients, the EMI SE can be derived with the equations (1)-(8) in the main text.
